# Associations between single and multiple cardiometabolic diseases and cognitive abilities in 474 129 UK Biobank participants

**DOI:** 10.1093/eurheartj/ehw528

**Published:** 2016-11-15

**Authors:** Donald M. Lyall, Carlos A. Celis-Morales, Jana Anderson, Jason M. R. Gill, Daniel F. Mackay, Andrew M. McIntosh, Daniel J. Smith, Ian J. Deary, Naveed Sattar, Jill P. Pell

**Affiliations:** 1 Public Health, Institute of Health and Wellbeing, 1 Lilybank Gardens, University of Glasgow, Glasgow G11 5HE, UK; 2 BHF Cardiovascular Research Centre, Institute of Cardiovascular and Medical Sciences, University of Glasgow, Glasgow G12 8TA, UK; 3 Centre for Cognitive Ageing and Cognitive Epidemiology, University of Edinburgh, Edinburgh EH8 9JZ, UK

**Keywords:** Cognitive ability, Diabetes, Hypertension, Coronary artery disease, UK Biobank

## Abstract

**Aims:**

Cardiometabolic diseases (hypertension, coronary artery disease [CAD] and diabetes are known to associate with poorer cognitive ability but there are limited data on whether having more than one of these conditions is associated with additive effects. We aimed to quantify the magnitude of their associations with non-demented cognitive abilities and determine the extent to which these associations were additive.

**Methods and results:**

We examined cognitive test scores in domains of reasoning, information processing speed and memory, included as part of the baseline UK Biobank cohort assessment (*N* = 474 129 with relevant data), adjusting for a range of potentially confounding variables. The presence of hypertension, CAD and diabetes generally associated with poorer cognitive scores on all tests, compared with a control group that reported none of these diseases. There was evidence of an additive deleterious dose effect of an increasing number of cardiometabolic diseases, for reasoning scores (unstandardized additive dose beta per disease = −0.052 score points out of 13, 95% CI [confidence intervals] −0.063 to − 0.041, *P* < 0.001), log reaction time scores (exponentiated beta = 1.005, i.e. 0.5% slower, 95% CI 1.004–1.005, *P* < 0.001) and log memory errors (exponentiated beta = 1.005 i.e. 0.5% more errors; 95% CI 1.003–1.008).

**Conclusion:**

Cardiometabolic diseases are associated with worse cognitive abilities, and the potential effect of an increasing number of cardiometabolic conditions appears additive. These results reinforce the notion that preventing or delaying cardiovascular disease or diabetes may delay cognitive decline and possible dementia.

## Introduction

### Background

Cognitive decline impacts adversely on the quality of life of affected individuals and their families and can impair adherence to treatment of comorbid conditions.^1^^,^^2^ Anecdotally, individuals worry more about cognitive decline and dementia than any other condition as they age.^3^ Therefore, there is substantial interest in identifying modifiable risk factors to ameliorate cognitive decline.^4^^,^^5^

Individual cardiometabolic diseases such as hypertension, coronary artery disease (CAD) and diabetes have been shown to associate with impaired cognitive function in cross-sectional studies.^6–8^ Cardiometabolic conditions and cognitive decline may simply share common risk factors or the association may be causal. Impaired cardiovascular function may cause cognitive decline by inhibiting cerebrovascular blood flow (called ‘neurovascular coupling’^9^) possibly leading to hypoperfusion and the amyloid beta plaques that characterize Alzheimer’s disease (AD).^9^^,^^10^ Some authors have suggested that the association may reflect reverse causation due to people with poorer cognitive function being less likely to adopt health-promoting lifestyles.^2^^,^^11^ While this may be the case, longitudinal data have also shown cardiometabolic diseases to be associated with subsequent decline in cognition over time.^8^ While it is established that individual cardiometabolic diseases are associated with non-demented cognitive ability,^12^ it is unknown whether there is an additive effect from having more than one disease.

UK Biobank is a very large, general population cohort that recruited participants in middle to older-age, prior to the onset of frank dementia. The data collected at baseline included cognitive function tests and prevalent cardiometabolic disease as well as relevant confounders including mood disorder which has not been available in many previous studies. The current study aims to determine whether there is evidence of a potential additive effect of three cardiometabolic diseases: diabetes hypertension and myocardial infarction/angina (the latter two herein referred to as CAD) on cognitive outcomes in the UK Biobank cohort. This is an important question since there is a rising number surviving with CAD and, as obesity levels rise, more are also developing and living longer with diabetes.

## Methods

### Materials and procedure

We examined three tests that were included as part of the UK Biobank baseline cognitive assessment. The complete battery is detailed in an open-access baseline paper.^13^ The first of these was a task with thirteen logic/reasoning-type questions and a two-minute time labelled fluid intelligence in the UK Biobank protocol but hereafter referred to as verbal-numerical reasoning (http://biobank.ctsu.ox.ac.uk/crystal/field.cgi?id=20016). The maximum score was 13. The Cronbach alpha coefficient for these items has been reported elsewhere as 0.62,^14^ and the task shows reasonable reliability across on average 4.3 years in 4253 participants with repeat data (*r* = 0.65, *P* <0.001). The next task was a visual memory test called pairs matching (http://biobank.ctsu.ox.ac.uk/crystal/label.cgi?id=100030), where participants were asked to memorize the positions of six card pairs, and then match them from memory while making as few errors as possible. We refer to this test as the memory task from here on. Scores on the memory test are for the number of errors that each participant made, and higher scores are, therefore, worse. The memory test did not show good reliability in 19 017 participants with longitudinal data (*r* = 0.16, *P* <0.001); however, this may partly be due to slight floor effects where most participants made few-to-no errors on repeat assessment, and it will be a more informative test variable when measured at baseline. Finally, participants completed a timed test, measured in milliseconds, of symbol matching; similar to the common card game Snap (http://biobank.ctsu.ox.ac.uk/crystal/field.cgi?id=20023) which we refer to as reaction time. The reaction time task has been shown to have acceptable test–retest reliability over a mean interval of 4.3 years (*r* = 0.54, *P* <0.001) in 19 327 participants who underwent repeat assessment. The reasoning task was added to the participant assessment part-way through the baseline assessment phase and sample sizes for the tasks, therefore, vary.

As part of the baseline assessment, participants were asked whether a physician had diagnosed myocardial infarction, angina, stroke, hypertension or diabetes. We defined CAD as angina and/or myocardial infarction. Participants self-reported other conditions freely on a separate assessment screen (e.g. atrial fibrillation). Townsend deprivation indices were derived from postcode of residence.^15^ They provide an area-based measure of socioeconomic deprivation derived from aggregated data on car ownership, household overcrowding, owner-occupation and unemployment. Education was based on self-report of the highest qualification achieved and dichotomised into university/college degree or less. (We have conducted additional analysis and found that analysing the ‘education’ variable as a more fine-grained ordinal variable of ‘degree; A-levels/AS-levels; O-levels/GCSEs; CSEs; NVQ/HND/HNC; none’ made no difference to the final results; data available upon request.) Participants self-reported their ethnic group and we recoded this into white and non-white.^16^ Body mass index was measured by trained research staff. Participants removed their shoes and heavy outer clothing. Weight was measured, to the nearest 0.1 kg, using a Tanita BC-418 MA body composition analyser and height using a Seca 202 height measure. Body mass index (BMI) was derived from: weight (kg)/(height (m) × height (m)). Smoking was coded as never, previous, or current smoker based on self-report. Frequency of alcohol intake was recorded as never, special occasions only, 1–3 times per month, 1–2 times per week, 3–4 times per week, daily/almost daily. Participants self-reported whether they were on medication for cholesterol, high blood pressure or on insulin. The minority of participants that did not know or chose not to answer were removed.

### Analyses

We excluded participants who declined to provide data on cardiometabolic diseases at baseline, as well as participants who reported chronic neurological diseases that could directly affect cognitive function; such as dementia or stroke (see Supplementary material online, *Table S1*). We also excluded participants outside the formal UK Biobank age range of 40–70 years. The remaining participants were classified into mutually exclusive categories according to the number and type of cardiometabolic diseases they reported, including a common comparison group comprising participants with none of the cardiometabolic diseases.

The cognitive tests were treated as outcome variables in a series of linear regression models. Cognitive data that were not normally distributed were transformed using STATA v.13,^17^ which was used for all analyses. We applied a natural log transformation to the reaction time scores which were positively skewed and used the LN + 1 function to transform the pairs-matching error scores which were significantly zero-inflated and positively skewed. After these adjustments, all outcome variables were normally distributed individually and when adjusted for covariates; in any case the final results were similar when the data were re-analysed using non-parametric equivalent tests as a check.

First, we undertook a series of sub-group analyses, comparing participants in each of the disease categories with the common comparison group of no cardiometabolic disease. We then entered all participants in a single model in which the number of eligible cardiometabolic diseases was entered as a numerical variable (ranging 0–3). Each of these models was run: a base model (adjusted for age in years, sex and white/non-white ethnicity); a partially adjusted model (also adjusted for Townsend score, education and depression); and fully adjusted (also adjusted for smoking status, alcohol intake, cholesterol/BP/insulin medication use and BMI). The results are reported as unstandardized beta coefficients for reasoning scores and exponentiated beta coefficients for the log transformed memory and reaction time data, because the latter are easier to interpret. For example, an exponentiated beta coefficient of 1.00 would equate to no difference and 1.01 would equate to a 1% increase in reaction time. Because of the large sample size we elected to use *P* <0.001 as nominal significance. This *P* value is conservative also to partly offset the risk of multiple comparisons. We also report the variance explained by each model (*r*^2^).

As additional analyses, we also ran a more typical single multivariate model testing for associations where reported CAD, diabetes and hypertension were not in mutually exclusive groups; i.e. someone could be in all three groups. These results were very similar to our reported findings and are not shown but available upon request. With regard to collinearity, variance inflation factors for variables in the final models were acceptable (range = 1.02–1.54).^18^

## Results

Of the 502 649 UK Biobank participants 22 221 (4.4%) reported a neurological illness and were excluded, leaving 480 428 participants. Of those, 478 567 (99.6%) provided relevant cardiometabolic disease information. We removed 10 participants that were outside the formal UK Biobank age range of 40–70 years, as they inflated the age/cognitive score error bars. This left 478 557 participants, who had a mean age of 56.44 years (SD = 8.10), and of which 216 758 were male (45.3%), 422 210 were of white origin (88.5%) and 318 464 (32.8%) had a degree. In terms of depression, 18 249 self-reported history of this disease (3.8%). Descriptive statistics split by cardiometabolic group are shown in *Table 1*. There were 474 129 participants with reaction time data, 437 765 with memory data and 158 631 with reasoning data (the latter having been added part-way through assessment).

**Table 1:** Descriptive statistics by cardiometabolic disease grouping

	Control group (*N* = 333 296)	diabetes (*N* = 8070)	Hypertension (*N*=103 821)	CAD (*N* = 8633)	CAD + diabetes (*N* = 1120)	CAD + hypertension (*N* = 8308)	diabetes + hypertension (*N* = 12 682)	diabetes + CAD + hypertension (*N* = 2627)	*P* value
Age, mean (SD)	55.15 (8.14)	57.49 (7.99)	59.03 (7.20)	61.66 (6.35)	62.27 (6.05)	62.02 (5.88)	59.94 (6.79)	62.12 (5.62)	<0.001
Sex, male *N* (%)	140 237 (42.08)	4463 (55.30)	49 480 (48.05)	6103 (70.69)	871 (77.77)	5749 (69.20)	7485 (59.02)	1960 (74.61)	<0.001
Townsend score, mean (SD)	−6.26 (3.00)	−6.26 (3.38)	−6.26 (3.13)	−6.26 (3.25)	−6.26 (3.50)	−6.26 (3.41)	−6.26 (3.39)	−6.18 (3.51)	<0.001
Depression, *N* (%)	9502 (2.85)	490 (6.07)	6067 (5.84)	461 (5.34)	77 (6.88)	576 (6.93)	854 (6.73)	222 (8.45)	<0.001
Caucasian, *N* (%)	294 997 (88.80)	6370 (79.38)	92 186 (89.10)	7673 (89.14)	936 (84.25)	7438 (89.93)	10 413 (82.54)	2197 (84.08)	<0.001
Degree, *N* (%)	117 563 (36.59)	2306 (29.15)	28 308 (27.60)	1841 (21.69)	193 (17.72)	1556 (19.02)	2914 (23.38)	402 (15.70)	<0.001
Medication use, *N* (%)	9599 (2.89)	2335 (29.09)	36 591 (35.30)	1674 (19.50)	225 (20.13)	2387 (28.78)	4849 (39.29)	655 (24.94)	<0.001
Reasoning scores, mean (SD)	6.10 (2.15)	5.57 (2.26)	5.85 (2.13)	5.60 (2.14)	5.18 (2.25)	5.56 (2.09)	5.57 (2.23)	5.30 (2.15)	<0.001
Log transformed-reaction time score, mean (SD)	4.36 (0.19)	4.73 (0.20)	4.54 (0.19)	4.70 (0.19)	5.77 (0.20)	5.80 (0.19)	4.14 (0.20)	5.83 (0.20)	<0.001
Untransformed median (IQR)	531 (476–602)	555 (492–634)	547 (489–625)	559 (500–637)	578 (512–671)	563 (503–644)	563 (501–641)	575 (512–656)	<0.001
Log +1 transformed pairs matching errors, mean (SD)	5.98 (0.70)	5.70 (0.70)	5.26 (0.70)	5.16 (0.70)	5.28 (0.72)	4.91 (0.69)	4.95 (0.71)	4.81 (0.68)	<0.001
Untransformed median (IQR)	3 (2–5)	3 (2–6)	4 (2–6)	4 (2–6)	4 (2–6)	4 (2–6)	4 (2–6)	4 (2–6)	<0.001

CAD, coronary artery disease; SD, standard deviation; IQR, interquartile range.

### Reasoning

In the base model, participants with cardiometabolic disease had poorer reasoning scores than those without (*Table 2*). The unstandardized beta coefficients ranged from −0.207 to −0.835 and all were statistically significant. Adjustment for potential confounders attenuated the associations. The variance explained by each model was *r*^2^ = 0.06 i.e. 6%, (base), 0.15 (partial) and 0.16 (full). All but three associations remained statistically significant at *P* <0.001.

**Table 2:** Reasoning and cardiometabolic diseases

	**Base model**^a^				**Partially adjusted**^b^				**Fully adjusted**^c^			
	Beta coefficient	95% CI		*P* value	Beta coefficient	95% CI		*P* value	Beta coefficient	95% CI		*P* value
Hypertension only	−0.396	−0.473	−0.319	<0.001	−0.300	−0.374	−0.226	<0.001	−0.183	−0.258	−0.108	<0.001
CAD only	−0.207	−0.233	−0.181	<0.001	−0.123	−0.148	−0.098	<0.001	−0.107	−0.135	−0.079	<0.001
Diabetes only	−0.491	−0.572	−0.410	<0.001	−0.336	−0.413	−0.259	<0.001	−0.270	−0.348	−0.192	<0.001
CAD & diabetes	−0.835	−1.055	−0.615	<0.001	−0.608	−0.819	−0.398	<0.001	−0.431	−0.643	−0.218	<0.001
Hypertension & diabetes	−0.510	−0.592	−0.427	<0.001	−0.313	−0.392	−0.234	<0.001	−0.248	−0.328	−0.169	<0.001
Hypertension & CAD	−0.403	−0.467	−0.339	<0.001	−0.240	−0.302	−0.179	<0.001	−0.151	−0.215	−0.087	<0.001
Hypertension, CAD & diabetes	−0.687	−0.833	−0.542	<0.001	−0.444	−0.583	−0.305	<0.001	−0.287	−0.429	−0.146	<0.001

Beta values reflect the difference vs. healthy controls; groups are mutually exclusive so no participant is in more than one.

CI, confidence interval; CAD, coronary artery disease.

a Adjusted for age, sex and ethnicity.

b Adjusted for age, sex, ethnicity, Townsend score, depression and education.

c Additionally adjusted for smoking status, alcohol intake, medication use and body mass index.

### Reaction time

Participants with cardiometabolic diseases had longer response times than those without. All the associations were statistically significant in the base model, with the exponentiated beta coefficients ranging from 1.009 to 1.045 (i.e. 0.9–4.5% slowing). Adjustment attenuated the coefficients and all but three remained statistically significant (*Table 3*). The variance explained by each model was *r*^2^ = 0.11 i.e. 11%, (base), 0.12 (partial) and 0.12 (full).

**Table 3:** Log reaction time and cardiometabolic diseases

	**Base model**^a^				**Partially adjusted**^b^				**Fully adjusted**^c^			
	Beta coefficient^d^	95% CI		*P* value	Beta coefficient^d^	95% CI		*P* value	Beta coefficient^d^	95% CI		*P* value
Hypertension only	1.030	1.026	1.034	<0.001	1.027	1.023	1.031	<0.001	1.021	1.016	1.025	<0.001
CAD only	1.009	1.008	1.010	<0.001	1.007	1.005	1.008	<0.001	1.005	1.004	1.007	<0.001
Diabetes only	1.016	1.012	1.020	<0.001	1.012	1.008	1.016	<0.001	1.008	1.004	1.012	<0.001
CAD & diabetes	1.045	1.034	1.056	<0.001	1.038	1.027	1.049	<0.001	1.029	1.018	1.040	<0.001
Hypertension & diabetes	1.022	1.018	1.026	<0.001	1.017	1.013	1.021	<0.001	1.013	1.009	1.017	<0.001
Hypertension & CAD	1.031	1.027	1.034	<0.001	1.027	1.024	1.030	<0.001	1.022	1.018	1.025	<0.001
Hypertension, CAD & diabetes	1.042	1.035	1.049	<0.001	1.035	1.028	1.042	<0.001	1.029	1.021	1.036	<0.001

Beta values reflect the difference vs. healthy controls. CI, confidence interval; CAD, coronary artery disease.

a Adjusted for age, sex and ethnicity.

b Adjusted for age, sex, ethnicity, Townsend score, depression and education.

c Additionally adjusted for smoking status, alcohol intake, medication use and body mass index.

d Exponentiated.

### Visual memory

In the base model, isolated CAD, as well as diabetes plus hypertension were associated with significantly more memory errors vs. the control group (*Table 4*). Following full adjustment for potential confounders, these associations were significant plus that of hypertension plus CAD. The variance explained by each model was *r*^2 ^=^ ^0.03 i.e. 3% (base), 0.03 (partial) and 0.03 (full).

**Table 4:** Log memory error scores and cardiometabolic diseases

	**Base model**^a^				**Partially adjusted**^b^				**Fully adjusted**^c^			
	Beta coefficient^d^	95% CI		*P* value	Beta coefficient^d^	95% CI		*P* value	Beta coefficient^d^	95% CI		*P* value
Hypertension only	1.013	0.996	1.029	0.129	1.004	0.988	1.021	0.607	1.009	0.993	1.026	0.277
CAD only	1.015	1.010	1.021	<0.001	1.010	1.005	1.015	<0.001	1.017	1.011	1.023	<0.001
Diabetes only	0.999	0.983	1.014	0.853	0.988	0.973	1.004	0.140	0.988	0.972	1.004	0.141
CAD & diabetes	1.008	0.965	1.053	0.715	0.996	0.954	1.041	0.874	0.994	0.951	1.040	0.801
Hypertension & diabetes	1.032	1.015	1.048	<0.001	1.021	1.004	1.037	0.012	1.031	1.014	1.048	<0.001
Hypertension & CAD	1.014	1.000	1.027	0.043	1.002	0.989	1.016	0.727	1.019	1.005	1.033	0.007
Hypertension, CAD & diabetes	1.022	0.993	1.051	0.134	1.009	0.980	1.038	0.549	1.027	0.998	1.057	0.073

Beta values reflect the difference vs. healthy controls. CI, confidence interval; CAD, coronary artery disease.

a Adjusted for age, sex, and ethnicity.

b Adjusted for age, sex, ethnicity, Townsend score, depression and education.

c Additionally adjusted for smoking status, alcohol intake, medication use and body mass index.

d Exponentiated.

### Dose effect of disease number

In the fully adjusted model, there were clear and statistically significant dose relationships between the number of cardiometabolic diseases and reasoning scores, log memory errors and log reaction time scores. As the number of diseases increased from 0 to 3, reasoning scores significantly fell while log reaction time and log memory errors significantly increased (i.e. got worse; *Table 5*).

**Table 5:** Cognitive abilities and cardiometabolic disease count

	Reasoning scores				Log reaction time				Log memory errors			
	95% confidence intervals				95% confidence intervals				95% confidence intervals			
	Beta coefficient	Lower	Upper	*P* value	Beta coefficient^a^	Lower	Upper	*P* value	Beta coefficient^a^	Lower	Upper	*P* value
One disease	−0.125	−0.152	−0.098	<0.001	1.006	1.005	1.008	<0.001	1.014	1.009	1.020	<0.001
Two diseases	−0.200	−0.251	−0.149	<0.001	1.019	1.016	1.022	<0.001	1.022	1.011	1.033	<0.001
Three diseases	−0.288	−0.043	−0.147	<0.001	1.029	1.021	1.036	<0.001	1.027	0.998	1.057	0.071
Additive dose effect	−0.052	−0.063	−0.041	<0.001	1.005	1.004	1.005	<0.001	1.006	1.003	1.008	<0.001

Beta values reflect the difference vs. healthy controls; except for the ‘additive dose effect’ beta which shows the linear effect of having zero to three diseases. All associations are adjusted for age, gender, ethnicity, Townsend deprivation scores, depression, education, smoking intake, alcohol, medication for insulin/hypertension/cholesterol and BMI (‘fully adjusted model’). The beta coefficients for one/two/three disease groups use the no disease (control) group as referent.

a Betas for log reaction time and memory errors are exponentiated.

### Additional analyses

We re-ran all analyses having excluded 2283 participants with self-reported atrial fibrillation. This did not affect any of the final results. We additionally tested for interaction between dose increase in number of diseases (0;1;2;3), and sex on cognitive scores. The interaction terms for log memory errors (*P* = 0.025) and log reaction time (*P* = 0.828) did not attain statistical significance at our threshold of 0.001. There was a borderline non-significant interaction (based on our conservative alpha of 0.001) between gender and increasing diseases on reasoning scores (interaction *b* = −0.036, 95% CI = −0.059 to −0.012, *P* = 0.003), where males showed a slightly larger effect of disease count (beta = −0.049 vs. −0.034; see Supplementary material online, *Table S2*).

We tested for interaction between decade of age (40–50; 51–60; 61–70) and disease number on cognitive outcomes. There was a statistically significant interaction between age decile and disease number on log reaction time (interaction exponentiated beta = 0.9990, 95% CI 0.99984–0.99995, *P* <0.001) where younger participants showed a slightly larger effect of increasing cardiometabolic diseases (e.g. exponentiated beta in 40–50 age group =1.009, 95% CI = 1.007–1.010, *P* <0.001), compared with older participants (e.g. 61–70 age group exponentiated beta =1.005, 95% CI = 1.004–1.005, *P* <0.001). There were no significant interactions for log memory errors (*P* = 0.090) and reasoning (*P* = 0.813). *Table S2* shows the effects of increasing disease count, split by sex and also decade of age.

## Discussion

### Interpretation

In this large, general population study, the association between cardiometabolic disease and poor cognitive function (specifically processing and reasoning domains) extended beyond diabetes to other diseases of hypertension and CAD. Furthermore, there was a clear relationship whereby the potential impact was greater among those with more than one cardiometabolic disease. The association with cardiometabolic disease count was broadly linearly additive for reasoning scores and log memory errors, and potentially multiplicative for log reaction time slowing. The negative association with these cognitive measures was independent of age, gender, ethnicity, socioeconomic status, educational level, adverse lifestyle risk factors and comorbid depression. In our study, the magnitude of the effect was modest yet significant; for example having CAD plus diabetes resulted in a 0.8 decrement in reasoning scores out of a maximum score of 13. However, given that average ages of participants in different groups were around 50s to early 60s, these findings are nevertheless important and herald further worsening of cognitive function as mostly middle-aged individuals with exposure to multi-morbidity age.^8^^,^^19^ In clinical terms, our results provide encouragement over the potential to limit cognitive decline by better prevention or delaying of cardiometabolic diseases. Incident dementia rates have dropped by 20% in the last two decades, and this may be due to better cardiovascular management.^20^

Previous studies have shown than cardiometabolic diseases are associated with poorer cognitive performance, but this is the first demonstration of an additive effect of disease count so far as any of the authors are aware. Demonstrating that the direction of the association is from cardiometabolic disease to cognition is supported by the current findings, although it does not exclude the possibility that worse cognitive performance may confer risk of cardiometabolic disease through lifestyle behaviours.^2^^,^^11^ It is interesting that our findings are mostly significant even after correction for smoking, alcohol intake and BMI, as these lifestyle factors may be expected to explain part of the association between cognitive ability and cardiometabolic disease. Clinically, the recognition of dementia into older age can be somewhat weighted towards decline in memory, and this may lead to under-recognition of cognitive disorders in cardiovascular disorders.

Lack of statistical power is a common problem when undertaking sub-groups analyses. UK Biobank is very large; with around 500 000 participants. Therefore, in spite of being a general population cohort recruited in middle-age the numbers in each sub-group were higher than achieved in the majority of individual studies. The smallest sub-group was 1120 participants with CAD plus diabetes.

### Limitations

The UK Biobank participants are representative of the general population in terms of breakdown by age, sex, ethnicity and deprivation. However, as with all general population cohort studies, they are likely to be unrepresentative in terms of the prevalence of lifestyle risk factors and disease. A causal relationship between cardiometabolic disease and cognitive decline is plausible. However, both causation and reverse causation have been postulated. All effect sizes attenuated somewhat from the base models when adjusted for factors like deprivation, lower education and smoking behaviour, reflecting a degree of confounding (which we have attempted to control for statistically). Cardiometabolic disease may impact adversely on cerebrovascular blood flow and, therefore, cognitive function. Alternatively people with worse mental ability may be less likely to engage in healthy behaviours that protect against cardiometabolic disease.^2^^,^^9^ In a cross-sectional study, it is not possible to establish the temporal relationship between cardiometabolic disease and cognitive decline and, therefore, the direction of causation; the same limitation is also often true for prospective studies. UK Biobank does not have a measure of lifetime intelligence from before the onset of major cardiometabolic pathology and, therefore, cannot extrapolate the relative contributions of these. A recent study which included data from UK Biobank showed that presence of two or three cardiometabolic diseases (in this case diabetes, myocardial infarction or stroke) was associated with earlier and more prevalent mortality, and our findings may be affected by a degree of prodromal physical decline in those people.^21^

We did not correct for type-1 error in our findings e.g. with Bonferroni or False Discovery Rate corrections,^22^ because our *P* value was set very conservatively at *P* <0.001. However, the very large sample size is such that type-1 error is a still possibility in our findings. Focussing on the effect sizes, the differences between groups were often modest, however, are nevertheless important.

Participants stated whether their doctor had diagnosed them with various individual cardiometabolic diseases; however, we are unable to verify these—however, recent evidence in UK Biobank shows that self-reported illnesses generally tally with self-reported medications.^23^ If these diagnoses were in some way over or under-reported, this misclassification may slightly attenuate or strengthen the association with cognitive test scores.

We adjusted for socioeconomic deprivation with the Townsend index. This gives an area-based average of deprivation rather than an individual-level detailed metric of social deprivation but is nevertheless a robust indicator of risk. There were differences between groups in terms of age (range 55–62). We controlled for this statistically, however, covarying for age in years may not completely capture age-related differences.

We found somewhat inconsistent results with the memory errors score, e.g. where the linear disease number effect was significant, but the effect of having diabetes plus CAD plus hypertension vs. no diseases, was not. The effect sizes were relatively small. None of the cognitive tests not been externally validated with more widely used tasks of fluid intelligence, and it is unclear how sensitively they measure cognitive impairment. Our findings with the memory test may reflect a degree of type-1 error.

### Future research

The current study used only the baseline data from UK Biobank and was, therefore, cross-sectional in nature, albeit of a scale rarely seen. All participants are currently undergoing repeat cognitive testing (assuming they choose to attend) as part of an MRI assessment, and are being followed-up via linkage to record linkage to routine clinical records.^24^^,^^25^ Therefore, future studies could investigate the association between baseline cardiometabolic disease and cognitive decline over time as well as the association between cognitive impairment and long-term cardiometabolic and other outcomes.

### Summary

Among 474 129 participants in UK Biobank, cardiometabolic diseases (diabetes hypertension and CAD) were associated with poorer cognitive function in relation to *reasoning, information processing speed* and (less convincingly) *memory*. These associations were generally independent of potential confounders. In addition to each disease having independent associations, there were dose relationships whereby having more than one cardiometabolic disease was associated with greater impairments in the cognitive domains of reasoning and processing speed. Our findings highlight the potential importance for cognitive health of both primary prevention of individual cardiometabolic diseases (i.e. preventing or delaying cardiovascular pathology) and secondary prevention among people with one condition who are at risk of developing a comorbid disease. Given rising levels of multi-morbidity and public health concerns regarding rising levels of cognitive decline, our work has important implications for future research in this important area.

## Supplementary material

Supplementary material is available at *European Heart Journal* online.

## Supplementary Material

Supplementary Data
