# Supplementary material for: Associations between single and multiple cardiometabolic diseases and cognitive abilities in 474 129 UK Biobank participants
Source: Eur Heart J. 2016 Nov 15;38(8):577–83. doi: 10.1093/eurheartj/ehw528 (PMC5381595; doi:10.1093/eurheartj/ehw528)
Supplement: Supplementary Data [file ehw528_supp.zip › Supp_Table2.docx]

**Supplementary Table 2: additive dose effect test stratified by sex and age.**

|  | **Reasoning scores** | | | | **Log reaction time** | | | | **Log memory errors** | | | |
| --- | --- | --- | --- | --- | --- | --- | --- | --- | --- | --- | --- | --- |
|  |  | 95% confidence intervals | |  |  | 95% confidence intervals | |  |  | 95% confidence intervals | |  |
| Additive dose effect of diseases (0;1;2;3) | Beta coefficient | lower | upper | P-value | Beta coefficient* | lower | upper | P-value | Beta coefficient* | lower | upper | P-value |
| Sex |  |  |  |  |  |  |  |  |  |  |  |  |
| Females | -0.034 | -0.054 | -0.015 | 0.001 | 1.005 | 1.004 | 1.006 | <0.001 | 1.010 | 1.006 | 1.014 | <0.001 |
| Males | -0.049 | -0.063 | -0.035 | <0.001 | 1.005 | 1.004 | 1.006 | <0.001 | 1.003 | 1.000 | 1.006 | 0.040 |
| Age group |  |  |  |  |  |  |  |  |  |  |  |  |
| 40-50 years | -0.083 | -0.118 | -0.048 | <0.001 | 1.009 | 1.007 | 1.010 | <0.001 | 1.006 | 0.998 | 1.013 | 0.129 |
| 51-60 years | -0.068 | -0.088 | -0.047 | <0.001 | 1.005 | 1.004 | 1.006 | <0.001 | 1.005 | 1.001 | 1.009 | 0.015 |
| 60-70 years | -0.033 | -0.047 | -0.019 | <0.001 | 1.005 | 1.004 | 1.005 | <0.001 | 1.005 | 1.002 | 1.008 | 0.002 |

Each beta value reflects the difference between that group vs. healthy controls; groups are mutually exclusive so no participant is in more than one. All associations are adjusted for age, gender, ethnicity, Townsend deprivation scores, depression, education, smoking intake, alcohol, medication for insulin/hypertension/cholesterol, and BMI (‘fully adjusted model’). The beta coefficients for one/two/three disease groups use the no disease (control) group as referent. Betas for log reaction time and memory errors are exponentiated.
