# Supplementary material for: Associations between single and multiple cardiometabolic diseases and cognitive abilities in 474 129 UK Biobank participants
Source: Eur Heart J. 2016 Nov 15;38(8):577–83. doi: 10.1093/eurheartj/ehw528 (PMC5381595; doi:10.1093/eurheartj/ehw528)
Supplement: Supplementary Data [file ehw528_supp.zip › Supp_Table1.docx]

**Supplementary Table 1: Excluded (self-reported) diseases**

Brain cancer/primary malignant tumour

Brain haemorrhage

Brain/intracranial abscess

Cerebral aneurysm

Cerebral palsy

Chronic/degenerative neurological problem

Dementia/Alzheimer's disease/cognitive impairment

Encephalitis

Epilepsy

Head injury

Infection of nervous system

Ischaemic stroke

Meningeal cancer/malignant meningioma

Meningioma (benign)

Meningitis

Motor neurone disease

Multiple sclerosis

Neurological injury/trauma

Neuroma (benign)

Other demyelinating condition

Other neurological problem

Parkinson's disease

Spina bifida

Stroke

Subarachnoid haemorrhage

Subdural haematoma

Transient ischaemic attack
